# When less is more: Non-contrast head CT alone to work-up hypertensive intracerebral hemorrhage

**DOI:** 10.1016/j.jocn.2022.04.006

**Published:** 2022-04-18

**Authors:** Claire Chen, Sophia Girgenti, Dania Mallick, Elisabeth B. Marsh

**Affiliations:** Johns Hopkins School of Medicine, Department of Neurology, Baltimore, MD, United States

**Keywords:** ICH, Hemorrhage, Imaging, Hypertension, Prediction

## Abstract

Hypertension is a common cause of intracerebral hemorrhage (ICH). The work up typically involves neuroimaging of the brain and blood vessels to determine etiology. However, extensive testing may be unnecessary for presumed hypertensive hemorrhages, and instead prolong hospital stay and increase costs. This study evaluates the predictive utility of hemorrhage location on the non-contrast head CT in determining hypertensive ICH. Patients presenting with non-traumatic ICH between March 2014 and June 2019 were prospectively enrolled. Hemorrhage etiology was determined based on previously defined criteria. Chi square and Student’s t tests were used to determine the association between patient demographics, ICH severity, neuroimaging characteristics, and medical variables, with hypertensive etiology. Multivariable regression models and an ROC analysis determined utility of CT to accurately diagnose hypertensive ICH. Data on 380 patients with ICH were collected; 42% were determined to be hypertensive. Along with deep location on CT, black race, history of hypertension, renal disease, left ventricular hypertrophy, and higher admission blood pressure were significantly associated with hypertensive etiology, while atrial fibrillation and anticoagulation were associated with non-hypertensive etiologies. Deep location alone resulted in an area under the curve of 0.726. When history of hypertension was added, this improved to 0.771. Additional variables did not further improve the model’s predictability. Hypertensive ICH is associated with several predictive factors. Using deep location and history of hypertension alone correctly identifies the majority of hypertensive ICH without additional work-up. This model may result in more efficient diagnostic testing without sacrificing patient care.

## 1. Introduction

Parenchymal intracranial hemorrhages or intracerebral hemorrhages (ICH) represent only about 15% of all strokes yet result in significant morbidity and mortality. The 30-day mortality rate ranges from 35% to 52% [1], highlighting the importance of rapid diagnosis and management. In order for this to occur, proper identification of hemorrhage etiology is critical, given the cause of hemorrhage directly influences both initial treatment strategies and long-term prognosis. Causal factors include: hypertension (HTN), cerebral amyloid angiopathy (CAA), medications such as anticoagulants, systemic disease (eg. liver cirrhosis, thrombocytopenia), and underlying vascular lesions. Work-up to determine the underlying cause can range from a single noncontrast head CT, to more advanced forms of brain and vessel imaging (CTA, MRA, DSA), echocardiography to look for valvular abnormalities or evidence of longstanding hypertension (left ventricular hypertrophy (LVH)), malignancy screening, and laboratory values to screen for coagulopathies. In previous studies, factors such as hemorrhage location and a positive history of hypertension have been associated with hypertensive ICH. Given these associations and that hypertension is the most common etiology of ICH, representing greater than 50% of all cases [2], the traditionally more extensive work-up may not be necessary for all, or even most, patients and leads to increased costs, radiation exposure, potential complications, and prolonged hospitalization.

The ability to correctly classify the etiology of ICH using a simple diagnostic algorithm would help to alleviate the unnecessary use of resources. Prior studies have proposed algorithms to correctly classify ICH by etiology using demographics such as age and race, and risk factors including hypertension, smoking, and chronic kidney disease [3]. However, none have evaluated the predictive potential of the initial noncontrast head CT to identify a group in whom additional testing may not be needed, without significantly sacrificing diagnostic accuracy. The aim of this study is to create a predictive model for identifying hypertensive hemorrhage for individuals presenting with ICH to reduce unnecessary diagnostic tests without sacrificing patient care.

## 2. Methods

### 2.1. Study population

This study was approved by the Johns Hopkins Institutional Review Board. Data for all ischemic stroke and ICH patients admitted to our large, urban Comprehensive Stroke Center are entered into a clinical research database as part of quality assurance practices through Get With the Guidelines (c) to create a prospectively-collected stroke registry. Patients were first assessed on presentation to the Emergency Department by our institution’s acute stroke team, underwent neuroimaging using noncontrast head CT, and were admitted to the hospital for further work-up and management. Informed consent was not required for this study given its observational nature. All patients presenting with a non-traumatic ICH to our institution between March 2014 and June 2019 were enrolled. Subarachnoid hemorrhages and epidural/subdural hematomas were excluded. Data were collected regarding: patient demographics (age, race, sex), severity of presentation (NIH Stroke Scale (NIHSS) score [4], ICH score [5], neuroimaging characteristics (hemorrhage size, location, degree of white matter disease- CHS score [6], cerebral microbleeds), and medical variables (history of: atrial fibrillation, hypertension, hyperlipidemia, diabetes, coronary artery disease, smoking), along with outcome data (modified Rankin scores (mRS) [7] at discharge and 90 days).

### 2.2. Neuroimaging

All patients underwent a non-contrast head CT upon presentation to the Emergency Department. The vast majority of patients also underwent additional brain imaging with MRI and vascular imaging using either CTA, MRA, or conventional angiography (DSA) as determined by the treating physician. Imaging was reviewed by a board certified neuroradiologist to determine lesion location. Lesion volume on noncontrast head CT was calculated using a volumetrics package available within the Carestream platform [8] by a trained technician. Areas of surrounding edema were not included in total volume, but noted, along with signs of herniation and intraventricular involvement. Cerebral microbleeds and degree of white matter involvement (CHS score [9]) were calculated using MRI. Presence of aneurysm, cavernoma, arteriovenous malformation, or other vascular malformation was noted on MRI and vascular imaging. Imaging was reviewed by two independent reviewers (CC, SG) with 20% overlap during the training phase to allow for calculation of inter-rater reliability, which was high. Discrepancies were discussed until consistency was achieved.

### 2.3. Classification of hemorrhage etiology

Hospital records were also reviewed by 2 members of the study team, blinded to the imaging analysis, and ultimately by a board-certified vascular neurologist (EBM). Hemorrhage etiology was classified based on previously a proposed algorithm from the SMASH-U trial [10], utilizing included variables. Categories included: non-stroke (traumatic ICH, sub/epidural ICH, tumor), stroke with hemorrhagic transformation, structural lesion (vascular malformation), systemic disease (systemic cause other than anticoagulation, hypertension, or CAA), medication (anticoagulation-related), cerebral amyloid angiopathy, hypertensive, and undetermined.

### 2.4. Statistical analysis

Analyses were performed using STATA version 14 (College Station, TX). The ability of ICH location on non-contrast head CT to correctly classify hypertensive ICH was determined as the primary outcome variable. Lesions were classified as deep if they were located in any of the following areas: basal ganglia, caudate, thalamus, pons. Chi-squared analyses and t-tests were first used to evaluate the association between the previously specified variables and hypertensive hemorrhage etiology. Based on univariate analyses, significant variables (p < 0.05) were entered into a multivariable regression model to determine factors predictive of hypertensive hemorrhage. An ROC analysis to calculate an area under the curve (AUC) was performed using deep location as the variable of interest to predict hypertensive etiology. This was compared to subsequent analyses adding: 1) clinical history of hypertension, 2) presence of LVH as a surrogate marker for hypertension, and 3) presence of additional work-up: echocardiogram, MRI, vessel imaging to determine the benefit of supplementary tests. As a conservative measure, a secondary analysis was performed excluding patients who did not have vascular imaging performed as part of their work-up, as they may have been inappropriately classified due to a lack of additional testing.

## 3. Results

Three hundred eighty patients with non-traumatic ICH were admitted over the study period. The average age of the cohort was 67 years. Thirty-five percent were black; 49% were male (see Table 1 for full characteristics of the patient population). All 380 patients received an initial non-contrast head CT on admission along with laboratory studies screening for platelets and coagulation factors. At least some form of expanded work-up was performed for the majority of patients. Two hundred five (54%) patients underwent further brain imaging with MRI while 288 (76%) had vascular imaging (n = 105 MRA, n = 224 CTA; overlap with some individuals having both modalities). Two-hundred forty-two (64%) underwent an echocardiogram; 115 (30%) CT of the chest/abdomen/pelvis; and 44 (12%) repeat MRI 2–3 months after discharge.

Following SMASH-U criteria, based on work-up, hypertensive hemorrhage was the most common diagnosis (42%), followed by medication-related ICH (16%), stroke with hemorrhagic transformation (13%), structural lesion/vascular malformation (8%), cerebral amyloid angiopathy (6%), bleed due to systemic disease (5%), and lesion resulting from other non-stroke (2%) etiologies. 9.5% of cases were undetermined.

### 3.1. Factors associated with hypertensive ICH

For full results, please see Table 1. Deep location (p < 0.001), black race (p = 0.003), history of hypertension (p < 0.001), left ventricular hypertrophy (p < 0.001), admission blood pressure (p < 0.001), and renal disease (p = 0.007) were associated with hypertensive etiology. Cortical location (p < 0.001), history of atrial fibrillation (p < 0.001), positive urine toxicology (p = 0.001), anticoagulant use (p < 0.001), elevated INR (p < 0.001), and obtaining an initial MRI (p < 0.001) were associated with hemorrhages of other etiologies.

In multivariable regression models combining deep location, age, race, sex, history of atrial fibrillation, history of hypertension, left ventricular hypertrophy, positive finding on MRI, and anticoagulant use, only deep location (p = 0.002), and history of hypertension (p = 0.041) remained significant. (Table 2).

**Fig. 1.**
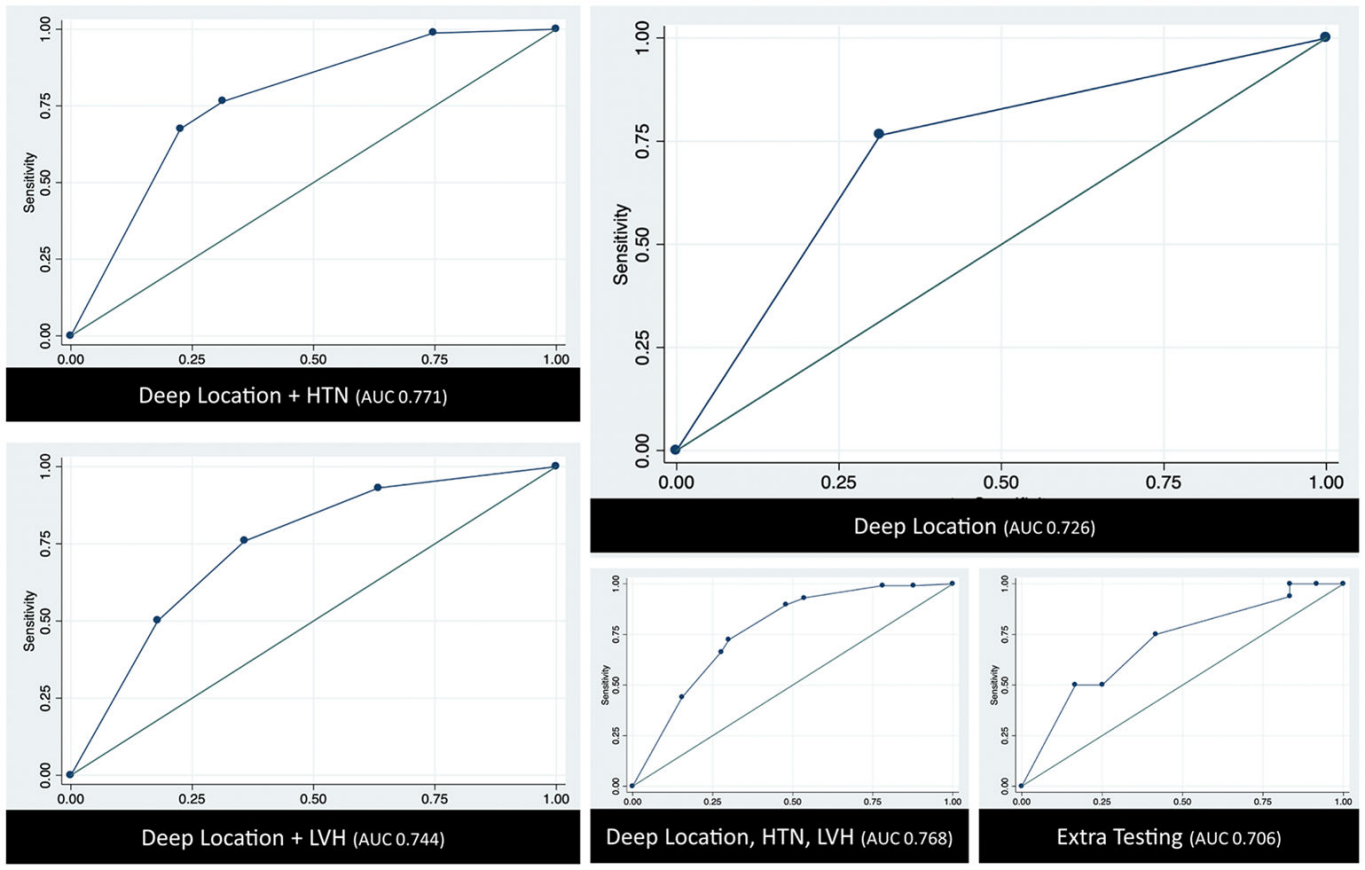
ROC Analyses for Models of ICH Etiology. Note that the AUC improves when history of hypertension or a surrogate marker (LVH) is added to deep location on CT; however, the addition of further variables does not significantly enhance performance.

When placed in an ROC analysis, the AUC using deep location to correctly predict hypertensive etiology was high (AUC = 0.726) (see Fig. 1). Adding LVH to the model improved the AUC to 0.744, but substituting known history of hypertension showed an even bigger improvement (AUC = 0.771). Results were not better when both hypertension and LVH were included (AUC = 0.768), or when other diagnostic tests (MRI, echocardiogram, urine toxicology, CT chest/abdomen/pelvis) were added to the model (AUC = 0.706). Results did not change when those without vascular imaging were removed from the analysis.

## 4. Discussion

Our results indicate that, while many factors are associated with hypertensive hemorrhage, including deep location on noncontrast head CT and a history of hypertension in the diagnostic model correctly identifies hypertensive ICH for the majority of patients without the need for additional work-up and without missing a significant number of cases. Including additional variables into the model did not increase its ability to correctly predict the diagnosis. This suggests that when a patient with known hypertension presents with a deep hemorrhage, more additional testing is not required to aid in diagnosis.

Consistent with prior studies, several variables were associated with hypertensive hemorrhage etiology in univariate analysis in addition to ICH location and history of hypertension including: left ventricular hypertrophy, black race, admission blood pressure, and renal disease. Both LVH and renal disease can be evidence of end-stage organ damage as the result of longstanding hypertension [11] and may be good surrogate markers in individuals who do not, or cannot, report a known history of disease. Prior studies have also reported that ICH due to hypertension often present with higher mean systolic blood pressure [12,13] though there is not sufficient evidence to use this variable alone as a diagnostic marker. Racial differences may be observed given the high rates of hypertension among the black population, with a higher rate of atrial fibrillation and anticoagulant use among white patients, which may predispose them to etiologies for ICH other than hypertension. This illustrates a potential opportunity to reduce risk of ICH by appropriately targeting specific risk factors in specific populations. Similarly, those factors associated with non-hypertensive etiologies have been described previously and illustrate the generalizability of our results. Anticoagulant use along with elevated INR value, and a positive urine toxicology, don’t preclude hypertension from being a contributing factor, but make other factors more likely.

After performing the multivariable analysis, the variables that remained significantly associated with the hypertensive etiology were deep location and history of hypertension. Further, deep location alone was a fairly good predictor of hypertensive ICH (AUC 0.726) that improved significantly when a history of hypertension was also considered (AUC 0.771). Previous studies have also shown that hypertension is associated with bleeds within the deeper parts of the brain, such as the basal ganglia, thalamus, caudate, and pons [14], and hypertension has been found to be twice as common in patients presenting with deep hemorrhages than in those with cortical hemorrhages. This may be not only that hypertension has a predilection for injuring deep perforating vessels, but that cortical bleeds are also more common places for embolic stroke with hemorrhagic transformation or amyloid angiopathy [15].

Given the importance of a history of hypertension in making the correct diagnosis, we explored the use of surrogate markers when reported history was unavailable. It is important to note that replacing a known history with the presence of LVH did improve the model, indicating that it may be useful surrogate, however, was not necessary when history of hypertension was available, and other markers such as creatinine were less useful.

Previous studies and models have revealed an association between deep hemorrhages and hypertensive etiology [16]. However, none have considered the predictability of hemorrhage location in determining the cause of an intracerebral hemorrhage. Other studies also noted associations with age and race, and risk factors including hypertension, smoking, and chronic kidney disease with hypertensive hemorrhage [3]. The DIAGRAM study group found that age, location, and small vessel disease (another potential marker for hypertension) were predictive of hemorrhage etiology [17]. We found that similar factors were associated with hypertensive ICH in our population; however, our study demonstrates that only 2 variables (history of hypertension and deep location) are needed to effectively predict ICH etiology, both of which can be gained upon presentation to the Emergency Department without advanced imaging.

Our study is not without limitations. It involves a cohort from a single Comprehensive Stroke Center, and though data were prospectively-collected, patients were not randomized so individual work-ups were based on provider preference. This may have resulted in a small portion of hemorrhages due to etiologies other than hypertension being mislabeled given that additional work-up was not obtained. However, as an academic training center, obtaining an MRI along with vascular imaging even when hypertension is thought to be the most likely etiology was common for the majority of patients admitted with nontraumatic ICH (76%), which allowed us to use advanced imaging as a ‘gold standard’ to aid in final diagnosis and determine the ability of other testing modalities to correctly classify patients. When the analysis was repeated using only patients who had undergone vascular imaging to rule out an underlying malformation or aneurysm, there were no significant changes to the results, indicating that our high AUC was not due to misclassifying individuals due to a lack of additional testing. In addition, patients were followed longitudinally both in-person, and when not possible, through chart review, making the likelihood of missing an alternative etiology such as an underlying malignancy, less likely. Within this cohort, there were no cases of rebleeding found and no alternative diagnoses not originally considered by the primary team and scheduled for additional work-up (eg., repeat MRI 3 months postdischarge) were noted. Finally, the number of patients presenting with ICH found to have an underlying vascular malformation was low. This may indicate a bias within our population given that we excluded those with SAH; however, this sample included all patients with intraparenchymal hemorrhage presenting to our Comprehensive Stroke Center, and the factors associated with hypertensive etiology were consistent with prior studies, indicating that our results are likely generalizable to other populations.

Despite the limitations, our findings indicate that a hemorrhage present in a deep location on noncontrast head CT, particularly in a patient with a known history of hypertension, is highly predictive of a hypertensive ICH and in this setting additional work-up is rarely helpful. It is also important to note that it does not miss a significant number of individuals, an important observation when considering decreasing the number of administered tests that may worsen your diagnostic accuracy. When history is not known, a surrogate marker, such as LVH on echocardiogram, can also be predictive.

While the model is highly predictive, it is imperfect, raising the question of what is the clinically acceptable threshold for missing an alternative cause. This may vary for each individual patient and the physician’s suspicion based on age or other variables, though we did not find any additional factors that were clearly suggestive within our cohort. Alternatively, as vascular malformation or aneurysm is the most feared alternative etiology, consideration of adding a CTA to the initial noncontrast head CT may be the most efficient way to mitigate risk and potentially add additional clinical benefit [18]. As we excluded those with subarachnoid hemorrhage within our cohort, we may have failed to see the true potential benefit of vascular imaging in our cohort. With or without vascular imaging, our model potentially allows for a more rapid diagnostic work-up, and limited exposure to radiation and potential complications, while minimizing costs and preventing delays in discharge due to need for additional testing.

## Figures and Tables

**Table 1:** Patient Characteristics.

		Hypertensive Etiology		
Variables	Overall Cohort	No (n=221)	Yes (n=158)	P- value
**Patient Demographics**				
Age, mean years (SD)	67.1 (15.9)	68.0 (1.1)	65.6 (1.2)	0.143
Sex, n male (%)	185 (48.7%)	99 (44.8%)	86 (54.5%)	0.064
Race, n black (%)	123 (34.7%)	59 (28.4%)	64 (43.8%)	0.003
**Severity of Presentation**				
NIHSS, mean (SD)	12.7 (9.5)	12.8 (0.9)	12.4 (0.9)	0.740
ICH Score, mean (SD)	1.8 (1.4)	2.0 (0.1)	1.6 (0.1)	0.048
**Neuroimaging Characteristics**				
MRI Performed, n (%)	205 (54%)	122 (59.51%)	83 (40.49%)	0.607
Vascular Imaging	288 (76%)	160	128	0.023
Performed, n (%)		(55.56%)	(44.44%)	
Hemisphere, n (%)				0.107
Right	166 (44.3%)	89 (41.2%)	77 (48.7%)	
Left	163 (43.5%)	95 (44.0%)	68 (43.0%)	
Bilateral	46 (12.3%)	32 (14.8%)	13 (8.2%)	
Circulation, n (%)				0.952
Infratentorial	55 (14.6%)	31 (14.3%)	23 (14.6%)	
Supratentorial	308 (81.9%)	179 (82.5%)	129 (81.7%)	
Multiterritorial	13 (3.5%)	7 (3.2%)	6 (3.8%)	
White Matter Grade, n (%)				0.421
1	8 (4.2%)	7 (6.3%)	1 (1.3%)	
2	58 (30.1%)	36 (32.1%)	22 (27.5%)	
3	58 (30.1%)	31 (27.7%)	27 (33.8%)	
4	38 (19.7%)	22 (19.6%)	15 (18.8%)	
5	15 (7.8%)	8 (7.1%)	7 (8.8%)	
6	6 (3.1%)	4 (3.6%)	2 (2.5%)	
7	9 (4.7%)	3 (2.7%)	6 (7.5%)	
8	1 (0.5%)	1 (0.9%)	0 (0.0%)	
Location, n (%)				<0.001
Deep	224 (59.4%)	96 (44.0%)	127 (80.4%)	
Cortical	111 (29.4%)	91 (41.7%)	20 (12.7%)	
Mixed	42 (11.1%)	31 (14.2%)	11 (7.0%)	
Herniation, n (%)	65 (17.2%)	44 (20.2%)	21 (13.3%)	0.081
Midline Shift, n (%)	161 (42.8%)	99 (45.6%)	62 (39.2%)	0.218
Edema, n (%)	269 (71.4%)	169 (77.5%)	100 (63.3%)	0.003
Intraventricular Involvement, n (%)	192 (50.9%)	107 (49.1%)	85 (53.8%)	0.367
Microbleeds, mean (SD)	3.42 (6.7%)	4.2 (0.8)	2.3 (0.6)	0.072
Volume, mean cc (SD)	30.68 (40.5%)	38.7 (3.3)	20.0 (1.9)	<0.001
**Medical Variables**				
Atrial Fibrillation, n (%)	62 (16.3%)	54 (24.4%)	8 (5.1%)	<0.001
Coronary Artery Disease, n (%)	70 (18.4%)	42 (19.0%)	28 (17.7%)	0.751
Diabetes, n (%)	92 (24.2%)	49 (22.2%)	43 (27.2%)	0.259
Drug use, n (%)	40 (10.5%)	25 (11.3%)	15 (9.5%)	0.570
Hyperlipidemia, n (%)	122 (32.1%)	72 (32.6%)	50 (31.7%)	0.848
Congestive Heart Failure, n (%)	35 (9.2%)	23 (10.4%)	12 (7.6%)	0.351
Hypertension, n (%)	287 (75.5%)	145 (65.6%)	142 (89.9%)	<0.001
Obesity, n (%)	30 (7.9%)	13 (5.9%)	17 (10.8%)	0.083
Prior Stroke, n (%)	90 (23.7%)	60 (27.2%)	30 (19.0%)	0.066
Smoking, n (%)	76 (20.0%)	51 (23.1%)	25 (15.8%)	0.082
Antiplatelet use, n (%)	150 (43.6%)	84 (41.0%)	66 (47.8%)	0.210
Anticoagulant use, n (%)	61 (18.1%)	58 (28.9%)	3 (2.2%)	<0.001
Urine Toxicology (cocaine), n (%)	14 (9.2%)	13 (17.3%)	1 (1.3%)	0.001
Left Ventricular	135	56	79	<0.001
Hypertrophy, n (%)	(55.8%)	(45.2%)	(67.5%)	
HDL, mean (SD)	53.65 (20.8%)	55.6 (2.1)	51.8 (2.0)	0.188
LDL, mean (SD)	87.97 (39.2%)	84.0 (3.3)	91.9 (4.2)	0.143
Hemoglobin A1c, mean (SD)	6.23 (1.4%)	6.2 (0.1)	6.2 (0.2)	0.990
Serum Glucose, mean (SD)	147.41 (61.9%)	148.3 (4.2)	145.9 (4.9)	0.712
Creatinine, mean (SD)	1.82 (6.5%)	1.7 (0.4)	2.1 (0.6)	0.547
Platelets, mean (SD)	223.84 (84.1%)	220.2 (5.9)	229.2 (6.4)	0.306
INR, mean (SD)	1.34 (1.0%)	1.5 (0.1)	1.1 (0.0)	<0.001
Admission Blood Pressure (Systolic), mean (SD)	163.6 (36.8)	155.8 (2.3)	174.7 (2.9)	<0.001
Admission Blood Pressure (Diastolic), mean (SD)	90.4 (24.0)	85.5 (1.4)	97.5 (2.1)	<0.001
BMI, mean (SD)	28.7 (7.4)	27.5 (0.5)	30.3 (0.6)	<0.001
**Outcome Data**				
Discharge mRS, n (%)				0.010
0	1 (0.3%)	0 (0.0%)	1 (0.6%)	
1	17 (4.5%)	14 (6.4%)	3 (1.9%)	
2	31 (8.2%)	15 (6.9%)	16 (10.1%)	
3	57 (15.1%)	33 (15.1%)	24 (15.2%)	
4	69 (18.3%)	35 (16.1%)	34 (21.5%)	
5	98 (26.0%)	49 (22.5%)	49 (31.0%)	
6	103 (27.4%)	72 (33.0%)	31 (10.6%)	
90 Day mRS, n (%)				0.239
0	10 (5.6%)	8 (7.9%)	2 (2.6%)	
1	13 (7.3%)	6 (5.9%)	7 (9.1%)	
2	21 (11.8%)	14 (13.9%)	7 (9.1%)	
3	26 (14.6%)	13 (12.9%)	13 (16.9%)	
4	15 (8.4%)	6 (5.9%)	9 (11.7%)	
5	26 (14.6%)	12 (11.9%)	14 (18.2%)	
6	67 (37.6%)	42 (41.6%)	25 (32.5%)	
Mortality, n (%)	104 (27.4%)	72 (32.6%)	32 (20.4%)	0.009

**Table 2:** Multivariable Logistic Regression Model.

Variable	Odds Ratio	95% Confidence Interval
Deep Location	9.18	1.01–83.84
Age	0.99	0.92–1.05
Race	1.02	0.20–5.11
Sex	1.53	0.28–8.37
Atrial Fibrillation	0.49	0.02–10.09
Hypertension	0.72	0.07–7.57
Tox Screen	0.08	0.01–1.54
Left Ventricular Hypertrophy	4.30	0.60–30.74
Positive MRI Findings	0.12	0.02–0.81
Prior Anti coagulation	1.00	–
